# Direct Control of Stem Cell Behavior Using Biomaterials and Genetic Factors

**DOI:** 10.1155/2018/8642989

**Published:** 2018-05-10

**Authors:** Jeong-Kee Yoon, Mi-Lan Kang, Joo Hyun Park, Kyoung-Mi Lee, Young Min Shin, Jin Woo Lee, Hyun Ok Kim, Hak-Joon Sung

**Affiliations:** ^1^Division of Cardiology, Severance Cardiovascular Hospital, Yonsei University College of Medicine, Seoul, Republic of Korea; ^2^Severance Biomedical Science Institute, College of Medicine, Yonsei University, Seoul, Republic of Korea; ^3^Department of Obstetrics and Gynecology, Yonsei University College of Medicine, Seoul, Republic of Korea; ^4^Department of Orthopaedic Surgery, Yonsei University College of Medicine, Seoul 120-752, Republic of Korea; ^5^Department of Laboratory Medicine, Yonsei University College of Medicine, Seoul, Republic of Korea; ^6^Department of Mechanical Engineering, Georgia Institute of Technology, Atlanta, GA, USA

## Abstract

Stem cells have recently emerged as an important candidate for cell therapy. However, some major limitations still exist such as a small quantity of cell supply, senescence, and insufficient differentiation efficiency. Therefore, there is an unmet need to control stem cell behavior for better clinical performance. Since native microenvironment factors including stem cell niche, genetic factors, and growth factors direct stem cell fate cooperatively, user-specified *in vitro* settings are required to understand the regulatory roles and effects of each factor, thereby applying the factors for improved cell therapy. Among others, various types of biomaterials and transfection method have been employed as key tools for development of the *in vitro* settings. This review focuses on the current strategies to improve stemness maintenance, direct differentiation, and reprogramming using biomaterials and genetic factors without any aids from additional biochemicals and growth factors.

## 1. Introduction

Stem cell therapy possesses significant advantages compared to conventional cell therapy using mature cells, as stem cells are more accessible and obtainable, are easy to culture and expand, and enable avoiding graft-versus-host rejection [1–3]. With such merits, stem cells have emerged as a candidate for cell therapy since 1968 when bone marrow transplantation surgery was conducted. Stem cells can self-renew and further differentiate into specific lineages upon stimulation. Among many kinds of stem cells, adult stem cells, represented by mesenchymal stem cells (MSCs), can be isolated or derived from many kinds of tissues and thus possess similar but different properties from each other. In a native microenvironment, MSCs are surrounded by stem cell niches composed of extracellular matrix (ECM) and growth factors. These microenvironment factors play instructive roles in directing stem cell behavior such as growth, lineage commitment, and stemness maintenance.

For clinical applications, stem cells have to be expanded because only a limited number of cells can be extracted from a tissue source. Moreover, when stem cells are expanded in a series of exhausted *in vitro* culture, the efficacy of their proliferation and differentiation decreases due to a progressive loss of stemness driven by senescence. To overcome such problems, state-of-the-art technologies using biomaterials, genetic factors, and growth factors which can mimic a native microenvironment or improve stem cell behavior have been employed recently. In conventional studies, various growth factors or cytokines were pretreated to stem cells during *in vitro* cultivation to induce a specific direction of differentiation for transplanting in a damaged tissue [4]. For example, fibroblast growth factor 2 (FGF2) has been reported to enhance MSC proliferation [5, 6]. The pretreated cells with growth factors, such as bone morphogenetic proteins (BMPs) or transforming growth factor *β* (TGF-*β*), can promote MSCs to differentiate into osteoblast or chondrocyte *in vitro* and induce efficient bone formation and cartilage regeneration compared to no treatment control [7–10]. However, FGF2 treatment is not able to overcome cellular senescence and the loss of differentiation potential of MSCs [11]. Moreover, because of the short half-life of growth factors, a large amount of growth factors is required to achieve the goal, resulting in high cost. Also, direct injection of growth factors may cause serious side effects such as osteophyte formation, swelling, and synovial hyperplasia [9]. Because of such disadvantages of growth factor treatment, applying biomaterials (e.g., natural, synthetic), biophysical factors (e.g., ultrasound), or biochemical factors (e.g., gene transfection) have emerged as alternative encouraging strategies to control stem cell fate.

**Figure 1:**
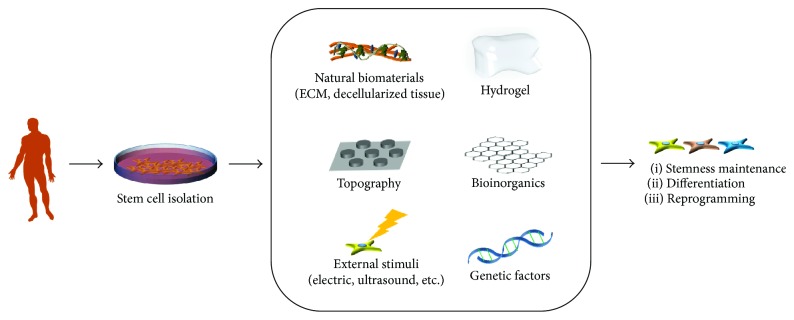
Strategies employing biomaterials and genetic factors to control stem cell fate. Stem cells can either maintain stemness, differentiate into specific lineages, or be reprogrammed to iPSCs.

Here, we review the current strategies to control stem cell fate using biomaterials, physiochemical factors, and genetic factors (Figure 1) in the absence of growth factor treatment. We first reviewed the strategies for stemness maintenance of adult stem cells using physiochemical factors (Table 1) and biomaterials (Table 2). Next, we introduced various types of biomaterials which can help adult stem cells to induce differentiation into specific lineages (Table 3). Finally, we reviewed genetic reprogramming methods for induced pluripotent stem cells (iPSCs) (Tables 4 and 5).

## 2. Improvement of MSC Stemness Using Biophysical Stimulation, Organic Compounds, and Biomaterials

Adult stem cells, represented by MSCs, are considered as an attractive agent for cell therapy because of their ability to self-renew and differentiate into various tissue cell types [12, 13]. However, the cell number when isolated is not usually sufficient for clinics. Therefore, a series of *in vitro* expansion of stem cell is indispensable. As MSCs lose their self-renewing ability and differentiation capacity during subculturing, maintenance of stemness has become an essential requirement for a successful stem cell therapy [14, 15]. Here, we review biophysical stimulation (Table 1), organic compound treatment (Table 1), and biomaterials (Table 2) as major methodological factors to maintain mature and homogeneous differentiation of stem cells [16, 17].

### 2.1. Biophysical Stimulation

Biophysical stimuli are one of important factors to enhance the differentiation capability of MSCs, for example, when a normal human cartilage was continuously exposed to physical pressure, such as joint loading. This stimulus went through cell membranes, thereby playing a pivotal role in structural maturation of cartilage. As another example, when MSCs were subjected to low-intensity pulsed ultrasound (LIPUS) stimuli *in vitro*, the cells differentiated into chondrocytes. Furthermore, when chondrogenic differentiation was induced in alginate, LIPUS-stimulated MSCs were not dedifferentiated even though the culture environment was not suitable for chondrogenic differentiation [18]. Along the same line, when MSCs were transplanted with PGA scaffold in a defect site post-LIPUS exposure for a week, the tissue morphology was maintained like an intact cartilage [19]. Another study demonstrated that when MSCs were stimulated with ultrasound, osteogenic differentiation was reduced compared to control MSCs [20, 21]. This result suggests that biophysical stimulation has significant effects on MSCs to keep the undifferentiated or differentiated status.

### 2.2. Biochemical Stimulation

MSCs express important pluripotent factors including Oct4, Sox2, Nanog, and cMyc, and these factors have been widely studied. However, expression of these factors reduces when MSCs undergo cell senescence during a series of subculture [22–24]. To address this issue, overexpression of pluripotent factors through lentiviral transfection was studied, thereby enhancing the self-renewal and differentiation potential of MSCs [25]. In addition to pluripotent factors, the telomere activity was found to reduce during *in vitro* cultivation. Thus, sirtuin 1 (SIRT1: a class III histone deacetylase protein) was treated to induce expression of telomerase reverse transcriptase (TERT) [26]. Sirt1 is also known as an important factor which regulates the lifespan, aging, metabolic homeostasis, and age-associated senescence of MSCs by controlling Sox2 acetylation [27].

In order to develop a better strategy to reduce cell senescence or to improve stemness, organic compounds are treated to MSCs to prevent the decrease of pluripotent marker expression. For example, since resveratrol is an antioxidant as well as Sirt1 activator, its treatment improved the stability of Sox2 by preventing acetylation and degradation of Sox2 [27]. Moreover, sustained treatment of resveratrol during ex vivo expansion maintained self-renewal and differentiation capacities from an early passage until a late passage [28]. These results suggest that treating stem cells with antioxidants can be a reliable option to maintain MSC stemness during subculture.

Another major cause of cell senescence is intracellular accumulation of reactive oxygen species (ROS) [29] (“oxidative stress”) which results in aging with end point apoptosis of MSCs [30, 31]. Nuclear factor (erythroid-derived 2)-like 2 (NRF2) plays a vital role in defending against oxidative stress at the cellular level. Therefore, conservation of NRF2 nuclear localization is important to overcome MSC aging during subculture [32, 33]. A previous study reported that treatment of t-BHQ, an antioxidant, increased translocation of NRF2 into the nucleus and prevented cellular senescence by regulation of the p53-Sirt1 axis, as p53 can suppress the transcriptional activity of Sirt1 by binding to the Sirt1 promoter [34]. Due to such changes in cellular behaviors under t-BHQ treatment, aged cells elevated the abilities for self-renewal and osteogenic differentiation [35]. Together, the results suggest that antioxidant treatment is a promising approach to reduce cell senescence especially in long-term culture for a successful MSC therapy *in vivo*.

### 2.3. Biomaterials

The extracellular matrix (ECM) controls stem cell fate (e.g., proliferation and differentiation) through integrin-receptor binding [36, 37]. Therefore, a series of biomaterials have been employed due to user-defined tunability of cell-matrix interaction (e.g., cell adhesion and cytoskeletal tension) as an artificial matrix platform. In this part, we introduce major types of biomaterials which have been used to maintain or to enhance stemness of adult stem cells, especially MSCs.

Biomaterials can be categorized into natural or synthetic materials in general. Among natural biomaterials, decellularized ECMs have been studied to control stem cell behavior recently. For example, the ECM where naive human MSCs (hMSCs) resided was decellularized and used for *in vitro* culture. This culture substrate was found to maintain stemness of human or mouse-derived adult stem cells most likely because it provided an *in vivo*-like stem cell niche [38–41]. Also, decellularized tendon tissue was found to maintain stemness and thereby promoted tenocyte differentiation of human tendon stem cells (hTSCs) by providing an amiable niche [42].

In addition to natural biomaterials, synthetic biomaterials have been recently designed to maintain or enhance stemness. For example, encapsulation of MSCs into hydrogels can mimic the three-dimensional microenvironment of native tissues. Polyacrylamide, alginate/GelMA, or pullulan-collagen hydrogels with low stiffness maintained stemness because they helped with the maintenance of low cytoskeletal tension [43–45]. Also, decreasing the cell matrix-binding affinity by reducing the Arg-Gly-Asp (RGD) density is revealed to enhance stemness in poly(carboxybetaine) hydrogels [46].

Besides, surface topography is a common method to control cell behavior related to stemness. While MSCs were cultured on nanopatterned poly(*ε*-caprolactone) (PCL) substrates with 120 nm pits in a square arrangement with a centre-centre spacing of 300 nm, stemness was enhanced [47]. On the other hand, nanotopography with an aligned shape (polydimethylsiloxane, 250 nm in depth, 350 nm in width, and with 700 nm pitch) did not enhance MSC stemness compared to a nontopographic surface [48].

**Figure 2:**
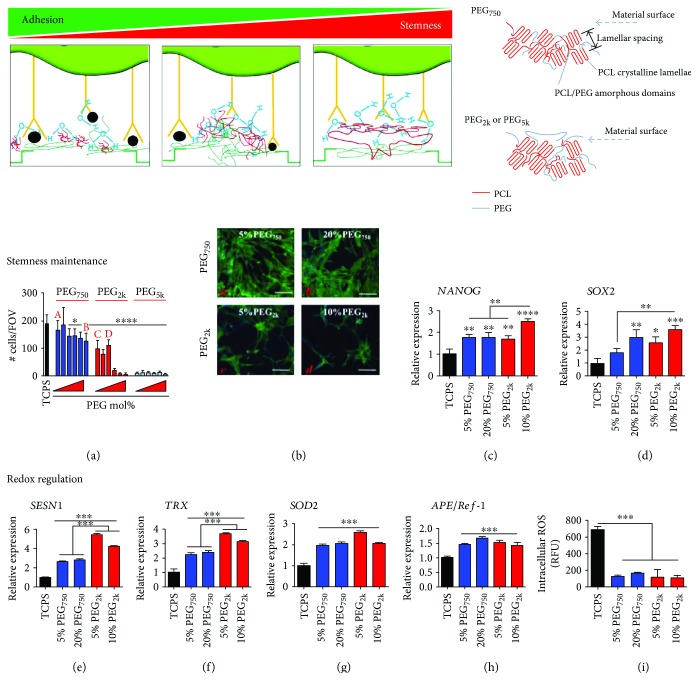
PEG chain length-dependent interactions with the PCL matrix enable stemness maintenance. Proper control of surface repellency by copolymerizing PEG_2k_ with PCL in a culture substrate form can improve stemness as cell-cell interaction increases relatively to cell-matrix interaction, thereby forming pseudo cell spheroids. Figure 2 is reproduced with permission from [52], American Chemical Society. All bars are mean ± S.D. ^∗^*p* < 0.05, ^∗∗^*p* < 0.01, ^∗∗∗^*p* < 0.001, ^∗∗∗∗^*p* < 0.0001 relative to TCPS or as indicated between the lines.

Polymeric surface coating serves as another promising option to control stemness. Poly-L-lysine (PLL) is a widely used polymer as a surface coating material, because it improves proliferation but retards replicative senescence by increasing the S-phase population of MSCs [49] and hematopoietic stem cells (HSCs) [50] in the cell cycle. Also, coating the culture substrate with PCL nanofibers which can mimic the size and shape of collagen fibers of ECM is also known to maintain stemness [51]. As another approach, poly(ethylene glycol)-poly(*ε*-caprolactone) (PEG-PCL) copolymers were used to control surface repellency by altering the molar percentage or chain length of PEG. This surface repellency induced aggregation of hMSCs by upregulation of cell-cell interaction proteins such as connexin-43, which further increased stemness with a significant decrease in intracellular ROS accumulation (Figure 2) [52].

Furthermore, nanofibrous scaffolds provided a 3D microenvironment to stem cells and thus enhanced stemness. For example, ASCs displayed an improved adhesion capacity with high rates of bioactivity and proliferation when cultured on emu oil-loaded nanofibers [53]. Mesenchymal stem cells (MSCs) showed a superior differentiation capacity towards typical mesenchymal lineages when grown in a nanostructured electrospun gelatin patch [54]. Especially, emu oil exhibited a free radical scavenging activity, thereby enhancing stemness [55].

Finally, a chitosan film induced spheroid formation and triggered a cell-cell interaction of hASCs, thereby enhancing stemness. After spheroid formation, the spheroid-forming hASCs expanded efficiently, formed a colony, and upregulated the expression of pluripotency marker genes compared to the monolayer-cultured control condition [56, 57].

In conclusion, the aforementioned types and formats of biomaterials were found to enhance or maintain stemness. We summarize the three major mechanisms by which the biomaterials enhanced or maintained stemness as follows: (1) reduction of cytoskeletal tension by reducing matrix stiffness, (2) spheroid formation by reduction of integrin-binding sites and consequent promotion of cell-cell interaction, and (3) antioxidative effects by radical scavenging activity. These strategies have potential to effectively improve stemness of MSCs in various biomaterial formats.

## 3. Direct Differentiation of MSCs Using Biomaterials

While most methods known to induce mesenchymal lineage differentiation of MSC depend on exposure to one or more soluble growth factors, a growing body of evidence suggests that it is possible to control MSC differentiation in the absence of soluble factors. MSCs exhibit the ability to differentiate towards specific lineages through biomaterials with modification of mechanical or biochemical properties, matrix composition, topography, and surface stiffness. This approach would simplify the tissue engineering procedure and be cost-effective. Here, we summarize recent studies that employed such approaches to induce direct differentiation of MSC via biomaterial technologies (Table 3).

### 3.1. Composition

Collagen and glycosaminoglycan (GAG), major components of natural ECM, play a key role in osteochondral regeneration. Hence, their combined (CG) scaffolds have been used successfully in tissue engineering applications for regeneration of cartilage and bone [57, 58]. A previous study reported the effect of the composition and stiffness of collagen and GAG scaffolds composed of chondroitin sulphate (CS) and hyaluronic acid (HA) on MSC differentiation [59]. The study showed that the lowest stiffness (0.5 kPa) of the CG scaffold facilitated chondrogenesis, while the stiffest (1.5 kPa) scaffold induced osteogenesis. This was the first evidence proving that osteochondral differentiation of MSC could be directed via scaffold composition using CG and further enhanced by the GAG type. When cellulose, another abundant natural polymer, was blended with silk at different compositions, growth and chondrogenesis of MSC were promoted [60]. This was also the first report demonstrating the potential of cellulose to induce chondrogenic differentiation of MSC.

Recent studies investigated whether chondrogenic differentiation of MSC could be directed by biomimetic or decellularized tissue-derived materials. Biomimetic polyacrylate substrates functionalized with the RGD integrin-binding peptide promoted chondrogenesis of MSC in the absence of any soluble growth factors [61]. They suggested that the amount of surface amine residues from the RGD peptide was a key regulator to inducing the differentiation. Several studies demonstrated that ECM components derived from the cartilage promoted chondrogenesis of MSC [62–64]. In all cases, however, the use of chondrogenic growth factors was found to be essential for MSC chondrogenesis with deposition of necessary matrix components. In addition, a study reported that novel particles derived from natural cartilage ECM induced chondrogenic differentiation of MSC even in the absence of TGF-*β* when the particles were used as a cell carrier [65].

Calcium phosphate- (CaP-) based ceramics play a significant role in bone repair due to their osteoconductive potential for filling lost bone volumes, when CaP nanoparticles and demineralized bone matrix (DBM) were fabricated as an injectable bone graft by incorporating polymerized high internal phase emulsions (polyHIPEs). This injectable bone graft was found to induce direct osteogenesis of MSC [58]. On the other hand, our recent study investigated the potential of 3D graphene substrates to induce spontaneous osteogenesis of MSC without additional stimuli [59]. These reports revealed that material-derived cues were able to guide MSC differentiation to osteogenesis in the absence of extrinsic biochemical inputs.

Proper regeneration of the myocardium is dependent on scaffold properties and thus can be enhanced by mimicking features of the myocardial ECM. A three-hydroxybutyrate and 3-hydroxyvalerate (PHBHV)/gelatin construct mimicking the myocardial ECM structure was developed to promote cardiac differentiation of MSC and cardiac resident cells without any chemical stimulation [66]. This study demonstrated that when specific physicochemical properties with a microtopograph were produced to mimic the structural and mechanical properties of myocardium in the PHBHV/gelatin construct, myogenesis of the stem cells was promoted. Electroconductive carbon nanotubes (CNT) demonstrated an ability to induce myogenic differentiation of MSC in the absence of additional stimuli [67]. Although the exact mechanism of this result is unclear, it was suggested that electrical stimulation of MSC by culturing on the CNT-based polylactic acid scaffold was a key factor to enhancing differentiation to cardiomyocytes.

### 3.2. Substrate Stiffness

Among the biophysical cues that were identified to regulate cell fate in static *in vitro* cultures, stiffness of culture substrates was suggested first as a key property in several important studies [60–64]. An increased stiffness of the culture substrate was found to induce osteogenic differentiation of MSCs [60, 61]. The role of matrix stiffness in directing lineage specification of MSCs was examined on the surface-charged methyl acrylate/methyl methacrylate (MA/MMA) polymer substrate with varying elastic modulus [60]. This study demonstrated that the substrate group with lower stiffness induced chondrogenesis of MSCs while the substrate with rigid stiffness induced osteogenic specification of MSCs. Although its specific mechanism is unclear, this study revealed that integrin *β*1 played a critical role in this process. Cells sense their mechanical microenvironment via integrin-ligand interactions which form focal adhesions and thereby regulate intracellular signaling [65]. Another study supported this finding [61] by showing that the soft (~0.5 kPa) substrate was effective in promoting neurogenesis of MSCs whereas the stiff (~40 pKa) one was effective in promoting their osteogenesis. Switching the biophysical microenvironment of MSCs from soft to stiff or stiff to soft substrates led to rewiring the two directions of MSC lineage specification.

**Figure 3:**
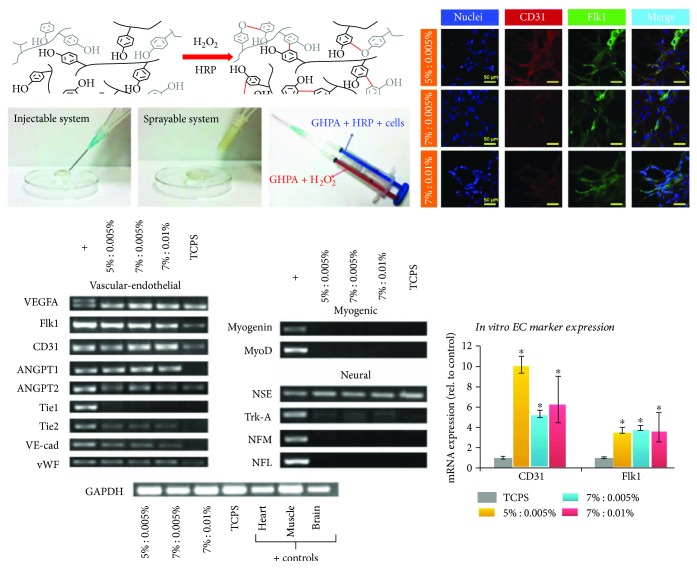
Gelation of GHPA by H_2_O_2_ and horseradish peroxidase-catalyzed cross linking. In vitro endothelial differentiation of hMSCs in GHPA hydrogels. Figure 3 is reproduced with permission from [63], John Wiley and Sons. ^∗^ indicates *p* < 0.05 in comparison to the control MSCs on tissue culture plate.

Although the differentiation potential of MSCs into endothelial cells (ECs) remains unclear [68, 69], some studies reported possible approaches to differentiate MSCs into ECs [63, 64]. A previous study demonstrated that a 3D matrix with tunable properties directed the differentiation of MSC towards vascular cell types [64]. We also applied in situ cross-linkable gelatin hydrogels by conjugating enzymatically cross-linkable hydroxyphenyl propionic acid (GHPA) (Figure 3) [63]. The 3D culture of MSCs in these hydrogels induced vasculogenesis both *in vitro* and *in vivo*. Our results showed that GHPA hydrogels induced spontaneous endothelial differentiation of MSC without any soluble factors.

### 3.3. Surface Topography

When cells are cultured on biomaterial substrates, surface topography is known as a key regulator of cell behavior. Several previous studies reported that surface topographical cues induced direct lineage specification of MSCs [66, 67, 70–72]. Microfeatures (40 *μ*m line, 20 *μ*m spacing, and 1 *μ*m height) of fibronectin strips printed on a poly(lactic-co-glycolic acid) (PLGA) thin film were found to direct linage commitment of MSCs [66]. In this study, modification of MSC morphology and cytoskeletal arrangement on the patterned film resulted in both neuronal and myogenic lineages, even if myogenic differentiation was dominant when the expression of functional proteins was examined. Along the same line to direct MSC differentiation, Lee et al. fabricated pseudo-3D microwells by templating a hydrazine-immobilized polyacrylamide gel displaying inverse features of circular surface topography via PDMS stamps (circular) [67]. As a result, small circular islands induced an adipogenic phenotype of MSCs while anisotropic geometries induced neurogenic differentiation. Micro- and nanostructured titanium surfaces were used as potential topographical cues to induce osteogenic differentiation of MSCs [71, 72]. The nanotubule-shaped titanium oxide surface structures, which have 70 to 100 nm titanium oxide nanotube arrays on them, induced cytoskeletal stress and thereby directed osteogenic differentiation of MSCs [71]. It was also reported that osteoblastic differentiation of MSCs was induced on the microstructured titanium surface (Ra = 3.22 *μ*m) through *α*2*β*1 integrin-mediated interactions with cocultured osteoblasts [72]. Our recent study determined causative roles of topographical cues in directing lineage specification of MSC via patterned graphene surfaces with additional evaluation of electrical stimulation as another cue [70]. Our result showed that expression of osteoprogenitor markers was increased by either (un)patterned graphene substrate or electrical stimulation while the expression of osteoblast makers was increased only when electrical stimulation was applied together with the surface patterns.

## 4. Selection of Genetic Factor and Source Cell Type for iPSC Reprogramming

Induced pluripotent stem cells (iPSCs) were introduced in 2006, which opened a new avenue for stem cell research and regenerative medicine [73]. Obtaining an adequate amount of stem cells is a major limitation for stem cell therapy and research. Previously, classical methods employed to induce pluripotency of somatic cells include somatic cell nuclear transfer (SCNT) and cell fusion. However, limitations associated with oocyte supply, low reprogramming efficiency, and phenotypic abnormalities of the produced animal offspring still hamper the widespread distribution of these classical methods [74].

Resident stem cells in various tissues were also studied as a promising source of stem cells, but the lack of appropriate markers to define their phenotypes and their low differentiation potential were considered as major hurdles for using these cell sources. Hence, the breakthrough idea of reprogramming somatic cells with ectopic pluripotent markers (Sox2, Oct3/4, Klf4, c-Myc, and Lin28) to eventually represent embryonic stem cell (ESC) characteristics was undisputedly attractive [73]. However, it is still controversial whether iPSCs possess the same pluripotency and differentiation ability to ESCs. Donor cell-specific epigenetic signatures remain even after reprogramming and thus generate problematic variations from the expected quality and characteristics of iPSCs in terms of homogeneity and the potential for maturation in stem cell therapy [75, 76]. Moreover, abnormalities created during the process of iPSC reprogramming, such as stablishing aberrant DNA methylation patterns, were found to increase the heterogeneity in iPSCs [77, 78]. Additionally, donor-specific genetic variations further increase the heterogeneity of iPSC genetic profiling, such as stablishing aberrant DNA methylation patterns.

### 4.1. Choice of Vectors for iPSC Reprogramming

Substantial progress has been made in the methodologies to improve the efficiency and efficacy of reprogramming somatic cells to iPSCs in the past decade (Table 4). The type of vectors used to overexpress ectopic pluripotency factors within the target cells are classified into integrating DNA vectors and nonintegrating DNA free vectors. Integrating vectors are further subclassified into insertional vectors including viral and linear DNA delivery systems whereas insertion-free transgene vectors include PiggyBac transposon [79, 80] and plasmid/episomal vectors. Recently, nonintegrating systems involving direct protein or microRNA vectors as well as various small molecules are used for reprogramming of somatic cells into iPSCs [81, 82]. Integrating DNA vectors represent an early generation tool for reprogramming and are still commonly used in experiments owing to their high efficiency. Combinations of retroviral or lentiviral Sox2, Oct4, Klf4, c-Myc, or Lin28 were most popularly used with or without the use of transgene selection markers. Especially, these first-generation viral vectors possess the potential for random insertional mutagenesis, but their undeniable high efficiency still renders them useful for a wide range of iPSC research. Such random insertional mutagenesis contributes to the unpredictability of iPSCs upon in vivo transplantation. Viral promoter-driven fluorescence and cre-LOX expression systems have been used to track and control ectopic gene expression but still generate insertional mutagenesis [83]. In order to overcome problems associated with mutagenesis resulting from ectopic gene insertion, adenoviral, episomal, or plasmid vectors were used as alternatives in the course of developing the next generation of reprogramming methods [84, 85]. However, although these alternatives were less prone to mutagenesis associated with ectopic gene integration, the major hindrance was the poor transfection efficiency, displaying efficiencies 1000–10,000 folds lower than those of conventional viral vectors. Further improvement in the reprogramming efficiency was achieved by applying non-DNA methods using Sendai viral vectors, small molecules, Lipofectamine, or miRNA transfections [86–90]. For example, cell membrane-penetrating proteins were tagged with Oct4, Sox2, Klf4, and c-myc for intracellular delivery and cell reprogramming. Human immunodeficiency virus transactivator of transcription (HIV-TAT) protein or polyarginine-tagged pluripotency factors were used to derive mouse and human iPSC lines. However, its low reprogramming efficiency still remains as a major hurdle to overcome [91]. Together, the aforementioned nonintegrating methods are promising to significantly reduce mutagenesis, but their reprogramming efficiencies need to be enhanced further as the efficiencies are still considerably lower (0.001%–) compared to integrating vectors (0.1%–1%). Generating transgene-free iPSCs serves as an attractive alternative because it can compensate for the low transfection efficiency. Addition of small molecules including histone deacetylase (HDAC) inhibitors and other epigenetic modifiers have been reported as representative examples [92, 93]. On the other hand, the number of ectopic vectors could be reduced by introducing supplementary compounds, where inhibitors of G9a histone methyltransferase could replace either Sox2, Oct4, or c-Myc during reprogramming of neural progenitor cells (NPCs) and fibroblasts in mice [94]. The TGF-*β* receptor antagonist also significantly increased the reprogramming efficiency and kinetics in murine [95, 96] and human fibroblasts [97].

It needs to be investigated further whether the type of vectors used to induce pluripotency contributes to the heterogeneity of produced iPSC lines or not. When retrovirus, Sendai virus, and episomal vectors were used for iPSC generation, different reprogramming strategies were applied to obtain human iPSCs. As a result, very similar global gene expression profiles were displayed but subtle differences were observed in the levels of gene expression, indicating that the heterogeneity of produced iPSC lines resulted from clonal signatures rather than the reprogramming method itself [98]. However, even when iPSCs were generated from cells of the same donor, characteristic aberrations in DNA methylation at the epigenomic level were shown to be dependent on the choice of reprogramming factors [99].

As a summary, random DNA aberrations are most notably caused by viral genome integration, leading to iPSC heterogeneity and unpredictability. Thus, nonintegrating systems should be primarily considered as a basic means for differentiation strategies towards clinical applications.

### 4.2. Donor Cell Characteristics and Stemness

The origin and quality of donor cells are also important factors to ensure successful reprogramming results. In particular, easy access, abundant quantity, and enough replenishment of donor cells after harvesting should be considered when the target donor source is selected.

**Figure 4:**
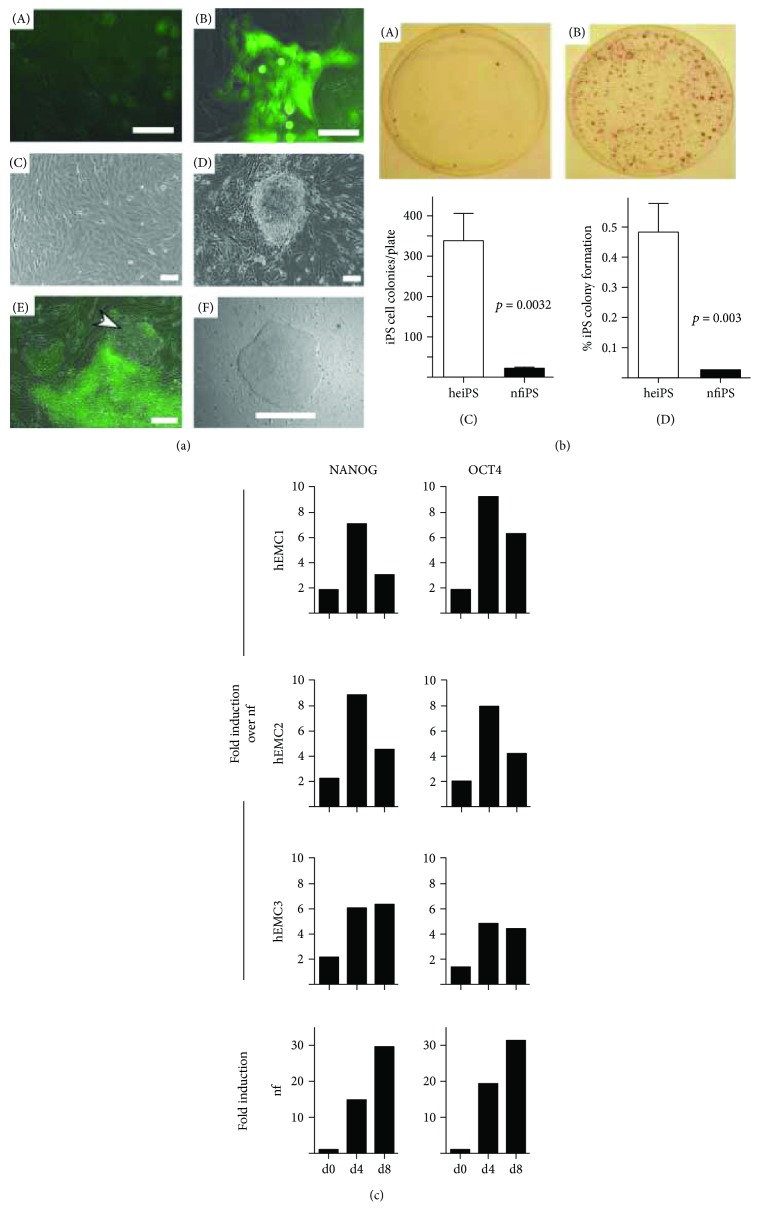
Pluripotency reprogramming of human endometrial cells (hEMC). hEMC-derived iPS (heiPS) showed higher expression of pluripotent markers compared to neonatal fibroblasts.  Figure 4 is reproduced with permission from [100], Oxford University Press.

Donor somatic cell-specific transcriptional and epigenetic signatures significantly contribute to the heterogeneity of efficiency and efficacy in reprogramming and differentiation of iPSCs (Table 5). The most widely used cell source for reprogramming into iPSCs is dermal fibroblasts, most frequently harvested from neonates as well as adults [73]. Keratinocytes (a type of bone marrow cells), peripheral blood cells (a type of CD34^+^ peripheral blood mononuclear cells), amniotic fluid cells, cord blood stem cells, endometrial stromal fibroblasts, and dental pulp cells have been reported as reliable sources of somatic cells for reprogramming (Figure 4) [100–106]. Moreover, it has been reported, even in cells which were originated from the same donor but from different organs, that the tissue-specific epigenetic signatures affect the heterogeneity of efficiency and efficacy in reprogramming and the differentiation potential of iPSC lines. Such observations were prominent in the early passages when the reprogramming process is not yet complete [107–109].

If stemness is enhanced, the negative effect of tissue-specific epigenetic signature may be attenuated during the process of serial passaging while losing the epigenetic memory sequentially. Characteristic DNA methylation patterns are originated from the donor cells and thus can be tracked in specific iPSC clones. Consequently, limitations in stemness of iPSCs as opposed to the full pluripotency of embryonic stem cells are inevitable. Experimental techniques to overcome the gap between the epigenetic memory of the donor cell and the stemness of the derived iPSC lines have been described in previous studies [110, 111]. One strategy is to increase the iPSC passage number while another approach is to introduce chromatin-modifying substances, which diminishes the epigenetic memory and enhances stemness. The process of acquiring pluripotency may not be complete upon immediate silencing expression of exogenous pluripotency factors but may continue for several rounds of cell passaging. iPSCs exhibit considerable differences in their telomere length and the global pattern of transcription and DNA methylation [110, 112, 113]. On the other hand, transgenes are usually silenced in the process of reprogramming by de novo methylation. When this process is not fully accomplished, gaining the pluripotency of the reprogrammed cells primarily relies on the exogenously introduced factors. When the endogenous pluripotent genes are halted from being fully expressed, these colonies are defined as “partially reprogrammed.” Within such colonies, pluripotency is frequently not fully acquired even after the exogenous factors are eventually turned off [114, 115]. Conversely, when ectopic transgenes are not silenced and exposed to residual activities or reactivation of the viral transgenes in the iPSC cells, tumor formation occurs as demonstrated in chimera experiments [73]. Other potential causes of epigenetic differences have also been attributed to either aberrant or incomplete reprogramming or even by various cell culture conditions [77, 116–119].

## 5. Conclusion

Although a growing body of evidence suggests stem cells as a promising candidate for cell therapy in the position of replacing somatic cells, the aforementioned issues regarding senescence and low differentiation efficiency must be addressed for successful clinical applications. In this review, we introduced state-of-the-art methods which are currently approached to improve efficiency and efficacy of stemness maintenance, direct differentiation, and iPSC reprogramming, with the minimal use of expensive and side effect-occurring growth factors. Biophysical stimulation, organic compound treatment, genetic transfection, and various types of biomaterials were employed to achieve the purposes. In particular, the effects of matrix stiffness, improving cell-cell interaction, and antioxidant treatment became a major part of interest. Additionally, biomaterials with specific composition, stiffness, and topography can serve as a promising toolbox to guide direct differentiation of stem cells. Finally, several combinations or individual uses of genetic factors to induce reprogramming of somatic cells were introduced as a means of generating iPSCs. Pros and cons of major reprogramming methods were discussed as well. Taken together, selection of biomaterials or other external factors needs to be customized for target-specific developments and application of stem cell therapy towards successful clinical applications.

## Figures and Tables

**Table 1:** Maintenance of stemness using biophysical and biochemical stimulations.

Type of stimulation	Details of condition	Type of cells	Observation	Ref.
Biophysical stimulation	Low-intensity pulsed ultrasound (LIPUS)	hMSCs	hMSCs differentiated into chondrocyte without dedifferentiation in nonchondrogenic differentiation environments.	[18]
	LIPUS	hMSCs	The transplanted cells differentiated into chondrocytes and regenerated defect sites of recipient cartilage.	[19]
	Ultrasound	hMSCs	Ultrasound treatment enhanced fracture healing by promoting osteogenic differentiation of hMSCs.	[20]
	Fluid flow	Osteocyte, osteoblast, and hMSCs	Flow stimulation promoted recruitment, proliferation, and differentiation of osteoprogenitor cells.	[21]

Overexpression of genetical factor	SRY- (sex-determining region Y-) box 2 (SOX2) Sirtuin 1 (SIRT1)	hMSCs	Overexpression of Sox2 enhanced stemness of MSCs during in vitro cultivation.	[23]
		hMSC	Overexpression of SirT1 prevented age-associated senescence of MSCs via Sox2 regulation.	[26, 27]
	Octamer-binding transcription factor 4 (Oct4) or pron. nanOg (Nanog)	hMSC	Viral transfection of Oct4 or Nanog enhanced the self-renewal and differentiation potential of MSCs.	[24, 25]

Treatment of organic compound	Resveratrol	hMSCs	Resveratrol treatment enhanced maintenance of the self-renewal and differentiation capacity of MSCs during ex vivo cultivation.	[28]
	Nuclear factor erythroid-derived 2-like 2 (NRF2)	hMSCs	Treatment of t-BHQ, the activator of NRF2, promoted self-renewal ability and osteogenic differentiation via inhibition of p53 expression.	[35]

**Table 2:** Maintenance of stemness using biomaterials.

Type of biomaterials	Details of materials	Type of cells	Observation	Ref.
Natural (nonsynthetic)	Decellularized ECM of undifferentiated hMSCs	hMSCs mASCs	Decellularized ECM of undifferentiated MSCs promoted self-renewal, colony formation, and stemness maintenance of hMSCs.	[38–41]
	Decellularized tendon tissue	hTSCs	Decellularized tendon tissues enhanced self-renewal and stemness maintenance of hTSCs.	[42]

Hydrogel	Polyacrylamide gels and PDMS stamps	hMSCs	Low cytoskeletal tension was maintained by controlling substrate stiffness as cell spreading was restricted, thereby enhancing stemness. Polyacrylamide gels and PDMS stamps were used to regulate biophysical parameters.	[43]
	Alginate/GelMA hydrogels	hBMSCs and GMSCs	Compared to alginate hydrogels, alginate/GelMA hydrogels maintained stemness due to decreased hydrogel stiffness.	[44]
	Pullulan-collagen hydrogel	mBMSCs	Biomimetic hydrogel maintained stemness of mouse bone marrow-derived MSCs (mBMSCs) compared to tissue plate culture, resulting in enhanced viability after in vivo injection.	[45]
	RGD-modified poly(carboxybetaine) hydrogel	hBMSCs	hMSCs formed 3D spheroids on the 5 *μ*M RGD substrate, and the stemness was well maintained compared to 5 mM RGD substrate, which enhanced osteogenic differentiation.	[46]

Topography	PCL	hMSCs	A surface nanopattern with 120 nm pits in a square arrangement with a center-center spacing of 300 nm enhanced stemness of hMSCs compared to the flat PCL surface.	[47]
	PDMS	hBMSCs	A PDMS nanopattern 250 nm in depth, 350 nm in width, and with 700 nm pitch decreased hBMSC stemness compared to the flat surface control.	[48]

Polymeric surface coating	PLL-coated surface	hBMSCs	PLL-coated surface improved proliferation but retarded the replicative senescence of hBMSCs by increasing the S-phase.	[49]
		hHSCs	PLL substrates increased the total number of hHSCs while stemness was maintained.	[50]
	PCL nanofiber	hMSCs	Bone marrow collagen-mimetic PCL nanofiber matrices increased the expression of self-renewal factors and cell-cell interaction markers in hMSCs.	[51]
	PEG-PCL copolymer	hMSCs	PEG-PCL copolymer exhibited moderate surface repellency and induced aggregation of hMSCs, which promoted stemness and lowered intracellular ROS accumulation.	[52]

Nanofibrous scaffold	Emu oil-loaded PCL/Coll nanofiber	hASCs	Emu oil-loaded nanofibers with higher tensile strength enhanced the expression of stemness, proliferation, and cell adhesion markers in hASCs compared to unloaded nanofibers.	[53]
	Gelatin nanofiber	hMSCs	3D culture of hMSC in a nanostructured electrospun gelatin patch maintained stemness of hMSCs for 3 weeks.	[54]

Chitosan	Chitosan film	hASC	The chitosan film induced spheroid formation of hASCs with higher activities of self-renewal and colony formation, as well as significant upregulation of pluripotency marker expression.	[56]
	Chitosan film + hypoxia	hUCBMSC	The chitosan film promoted spheroid formation of hUCBMSC under hypoxia than normoxia. HIF-1 additionally induced expression of stemness genes.	[57]

**Table 3:** Direct differentiation using biomaterials.

Property	Type of materials	Differentiation	Details of materials	Comments	Ref.
Composition	Scaffold	Chondrogenesis	Cellulose/silk blend	Growing MSCs on a specific blend combination of cellulose and silk in a 75 : 25 ratio significantly upregulated expression of chondrogenic markers.	[120]
		myogenesis	ECM-like porous scaffold of poly(3-hydroxybutyric acid-co-3-hydroxyvaleric acid) (PHBHV)/gelatin blends	PHBHV/gelatin constructs mimicking myocardial structural properties.	[121]
		Chondrogenesis/osteogenesis	Collagen-glycosaminoglycan	Collagen-chondroitin sulphate (CCS) scaffolds enhanced osteogenesis while collagen-hyaluronic acid (CHyA) scaffolds enhanced chondrogenesis.	[122]
		Cardiomyogenesis	Carbon nanotube/poly-L-lactide acid (PLA) nanofiber	The two-pronged carbon nanotube template provided a biomimetic electroactive cue, thereby directing MSC differentiation.	[13]
	Decellularized tissues	Chondrogenesis	Cartilage extracellular matrix-derived particles (CEDPs)	Microtissue aggregates (BMSCs and CEDPs (263 ± 48 *μ*m) cocultured in a rotary cell culture system) showed a more rapid restoration of joint functions with superior cartilage repair compared to the control groups in vivo.	[3]
		Osteogenesis	Calcium phosphate nanoparticles and demineralized bone matrix (DBM) particles incorporated into injectable polyHIPE	PolyHIPE compositions with BMSCs promoted osteogenic differentiation through upregulation of bone-specific marker expression compared to a time zero control.	[4]
	Bioinorganics	Osteogenesis	3D graphene foams (GFs)	3D GF culture platforms maintained stem cell viability and promoted osteogenic differentiation.	[123]
	Biomimetics	Chondrogenesis	Polyacrylate substrate functionalized with RGD peptide	Biomimetic polyacrylate substrates can direct chondrogenic differentiation of mMSCs, hMSCs, and mouse KSCs in the absence of exogenous TGF-bs.	[124]

Substrate stiffness	Hydrogels	Osteogenesis/neurogenesis	Polyacrylamide (0.5~40 kPa) hydrogel substrate	MSCs on soft (~0.5 kPa) gels promoted expression of neurogenesis markers while MSCs on stiff (~40 kPa) substrates elevated expression of osteogenesis markers. Transfer of MSCs from soft to stiff or stiff to soft substrates led to a switch in the lineage specification.	[60]
		Osteogenesis/chondrogenesis	Methyl acrylate/methyl methacrylate (18–72 MPa) hydrogel substrate	Both chondrogenic and osteogenic markers were elevated when MSCs were grown on substrates with stiffness < 10 MPa. MSCs on lower stiffness gels express elevated chondrogenesis markers while MSCs on the higher stiff substrates express elevated osteogenesis markers.	[61]
		Angiogenesis	Gelatin hydrogel conjugating enzymatically cross linkable hydroxyphenyl propionic acid (GHPA)	GHPA as a promising soluble factor-free cell delivery template induced endothelial differentiation of MSCs with robust neovasculature formation with favorable host responses.	[63]
		Angiogenesis	PEGylated fibrin 3D matrix	Endothelial differentiation of MSC was induced by the 3D PEGylated fibrin matrix.	[64]

Surface topograpy	Film	Neurogenesis/myogenesis	Micropatterned poly(lactic-co-glycolic acid) (PLGA) ultrathin film	Micropattering: microsize lanes of 20 *μ*m width separated by 40 *μ*m wide grooves on a PLGA ultrathin film (16.3 ± 1.5 *μ*m)	[66]
	Hydrogel	Adipogenesis/neurogenesis	Hydrazine-treated polyacrylamide gel (circular and anisotropic geometry)	Cells cultured in small circular islands show elevated expression of adipogenesis markers while cells that spread in anisotropic geometries elevated expression of neurogenic markers.	[67]
	Bioinorganics	Osteogenesis/neurogenesis	Graphene/electrical stimulation	Specific combinations of nonbiological inputs—material type, electrical stimulation, and physical patterns on graphene substrates regulated hMSC lineage specification.	[70]
		Osteogenesis	Nanotubule-shaped titanium oxide surface	Small (30 nm diameter) nanotubes promoted cell adhesion without noticeable differentiation, whereas larger (70 to 100 nm diameter) nanotubes elicited a dramatic stem cell elongation (10-fold increased), which induced cytoskeletal stress and selective differentiation into osteoblast-like cells.	[71]
		Osteogenesis	Titanium substrate	Surface microstructure and surface energy from microstructured Ti substrate were able to direct osteogenic differentiation of mesenchymal stem cells.	[72]

**Table 4:** iPSC reprogramming and type of gene transfection.

Type	Advantages	Disadvantages	Transgene expression	Efficiency	Ref.
Virus					
Adenovirus	Nonintegrative; infects dividing and nondividing cells	Low efficiency	No	0.0001~0.01%	[84, 85]
Lenti/retrovirus	Ease of handling with experience; medium–high efficacy	Integration of foreign DNA into genome; residual expression of reprogramming factors; controversy regarding tumor formation	Yes	0.1~1%	[73, 125]
Sendai virus	Medium–high efficiency; nonintegrating; robust protein-expressing property; wide host range	Involve viral transduction	No	0.5~1.0%	[88, 89]
Plasmid vector					
Episomal	Nonintegrative; simple to implement to laboratory setup; less time-consuming	Very low efficiency; the use of potent viral oncoprotein (SV40LT antigen)	No	3–6 × 10 − 6	[87, 126]
Minicircle	More persistent transgene expression; lack bacterial origin	Very low efficiency	No	0.01%	[127]
miRNA	Relative high efficiency; nonintegration; easily automated, making it an exciting candidate for routine biomanufacture.	Requires high gene dosages and multiple transfections; daily transfection; controversy in reproducibility and mitigating cost effectiveness	No	1.4~2%	[128, 129]
PiggyBac transposons	Elimination of insertional mutagenesis; no footprint upon excision; higher genome integration efficiency	Inefficient excision, potential for genomic toxicity	Excision with transposase	0.1~1%	[80]
Protein	Free of genetic materials; direct delivery of reprogramming factor proteins	Slow kinetics, low efficiency; difficulties in generation and purification of reprogramming protein	No	0.005~0.001%	[130]
Small molecules	Ease of handling; no requirements for reprogramming factors	More than one target, toxicity	No	0.3~0.5%	[86]

**Table 5:** iPSC reprogramming and donor cell type.

Donor cell type	Ref.
Adipose-derived stem cells	[131]
Amniotic fluid	[132]
Blood cell cord blood stem cells	[104]
B lymphocytes	[133]
Bone marrow cells	[134]
Cardiac myocytes	[135]
Dental pulp	[136]
Dermal fibroblasts	[137]
Endometrial stromal fibroblasts	[100]
Hematopoietic progenitor cells	[138]
Hepatocytes	[139]
Keratinocytes (from hair pluck)	[101]
Pancreatic *β*-cells	[140]
Peripheral blood mononuclear cell	[126]

## References

[1] Ringdén O., Uzunel M., Rasmusson I. (2006). Mesenchymal stem cells for treatment of therapy-resistant graft-versus-host disease.

[2] Polchert D., Sobinsky J., Douglas G. (2008). IFN-*γ* activation of mesenchymal stem cells for treatment and prevention of graft versus host disease.

[3] Yanez R., Lamana M. L., Garcia-Castro J., Colmenero I., Ramirez M., Bueren J. A. (2006). Adipose tissue-derived mesenchymal stem cells have in vivo immunosuppressive properties applicable for the control of the graft-versus-host disease.

[4] Koay E. J., Athanasiou K. A. (2009). Development of serum-free, chemically defined conditions for human embryonic stem cell–derived fibrochondrogenesis.

[5] Solchaga L. A., Penick K., Porter J. D., Goldberg V. M., Caplan A. I., Welter J. F. (2005). FGF-2 enhances the mitotic and chondrogenic potentials of human adult bone marrow-derived mesenchymal stem cells.

[6] Tsutsumi S., Shimazu A., Miyazaki K. (2001). Retention of multilineage differentiation potential of mesenchymal cells during proliferation in response to FGF.

[7] James D., Levine A. J., Besser D., Hemmati-Brivanlou A. (2005). TGF*β*/activin/nodal signaling is necessary for the maintenance of pluripotency in human embryonic stem cells.

[8] Sakaki-Yumoto M., Katsuno Y., Derynck R. (2013). TGF-*β* family signaling in stem cells.

[9] van Beuningen H. M., Glansbeek H. L., van der Kraan P. M., van den Berg W. B. (1998). Differential effects of local application of BMP-2 or TGF-*β*1 on both articular cartilage composition and osteophyte formation.

[10] Sailor L. Z., Hewick R. M., Morris E. A. (1996). Recombinant human bone morphogenetic protein-2 maintains the articular chondrocyte phenotype in long-term culture.

[11] Hellingman C. A., Koevoet W., Kops N. (2010). Fibroblast growth factor receptors in in vitro and in vivo chondrogenesis: relating tissue engineering using adult mesenchymal stem cells to embryonic development.

[12] De Becker A., Van Riet I. (2016). Homing and migration of mesenchymal stromal cells: how to improve the efficacy of cell therapy?.

[13] Lazarus H. M., Haynesworth S. E., Gerson S. L., Rosenthal N. S., Caplan A. I. (1995). Ex vivo expansion and subsequent infusion of human bone marrow-derived stromal progenitor cells (mesenchymal progenitor cells): implications for therapeutic use.

[14] Bruder S. P., Jaiswal N., Haynesworth S. E. (1997). Growth kinetics, self-renewal, and the osteogenic potential of purified human mesenchymal stem cells during extensive subcultivation and following cryopreservation.

[15] Ksiazek K. (2009). A comprehensive review on mesenchymal stem cell growth and senescence.

[16] Lam J., Lu S., Lee E. J. (2014). Osteochondral defect repair using bilayered hydrogels encapsulating both chondrogenically and osteogenically pre-differentiated mesenchymal stem cells in a rabbit model.

[17] Barry F., Boynton R. E., Liu B., Murphy J. M. (2001). Chondrogenic differentiation of mesenchymal stem cells from bone marrow: differentiation-dependent gene expression of matrix components.

[18] Lee H. J., Choi B. H., Min B. H., Park S. R. (2007). Low-intensity ultrasound inhibits apoptosis and enhances viability of human mesenchymal stem cells in three-dimensional alginate culture during chondrogenic differentiation.

[19] Park K., Cho K. J., Kim J. J., Kim I. H., Han D. K. (2009). Functional PLGA scaffolds for chondrogenesis of bone-marrow-derived mesenchymal stem cells.

[20] Padilla F., Puts R., Vico L., Guignandon A., Raum K. (2016). Stimulation of bone repair with ultrasound.

[21] Brady R. T., O'Brien F. J., Hoey D. A. (2015). Mechanically stimulated bone cells secrete paracrine factors that regulate osteoprogenitor recruitment, proliferation, and differentiation.

[22] Go M. J., Takenaka C., Ohgushi H. (2008). Forced expression of Sox2 or Nanog in human bone marrow derived mesenchymal stem cells maintains their expansion and differentiation capabilities.

[23] Yoon D. S., Kim Y. H., Jung H. S., Paik S., Lee J. W. (2011). Importance of Sox2 in maintenance of cell proliferation and multipotency of mesenchymal stem cells in low-density culture.

[24] Liu T. M., Wu Y. N., Guo X. M., Hui J. H. P., Lee E. H., Lim B. (2009). Effects of ectopic Nanog and Oct4 overexpression on mesenchymal stem cells.

[25] Ranzani M., Cesana D., Bartholomae C. C. (2013). Lentiviral vector–based insertional mutagenesis identifies genes associated with liver cancer.

[26] Chen H., Liu X., Zhu W. (2014). SIRT1 ameliorates age-related senescence of mesenchymal stem cells via modulating telomere shelterin.

[27] Yoon D. S., Choi Y., Jang Y. (2014). SIRT1 directly regulates SOX2 to maintain self-renewal and multipotency in bone marrow-derived mesenchymal stem cells.

[28] Yoon D. S., Choi Y., Choi S. M., Park K. H., Lee J. W. (2015). Different effects of resveratrol on early and late passage mesenchymal stem cells through *β*-catenin regulation.

[29] Harman D. (1956). Aging: a theory based on free radical and radiation chemistry.

[30] Zhu W., Chen J., Cong X., Hu S., Chen X. (2006). Hypoxia and serum deprivation-induced apoptosis in mesenchymal stem cells.

[31] Sart S., Song L., Li Y. (2015). Controlling redox status for stem cell survival, expansion, and differentiation.

[32] Surh Y.-J., Kundu J., Na H.-K. (2008). Nrf2 as a master redox switch in turning on the cellular signaling involved in the induction of cytoprotective genes by some chemopreventive phytochemicals.

[33] Zhu H., Zhang L., Itoh K. (2006). Nrf2 controls bone marrow stromal cell susceptibility to oxidative and electrophilic stress.

[34] Nemoto S., Fergusson M. M., Finkel T. (2004). Nutrient availability regulates SIRT1 through a forkhead-dependent pathway.

[35] Yoon D. S., Choi Y., Lee J. W. (2016). Cellular localization of NRF2 determines the self-renewal and osteogenic differentiation potential of human MSCs via the P53–SIRT1 axis.

[36] Kuhn N. Z., Tuan R. S. (2010). Regulation of stemness and stem cell niche of mesenchymal stem cells: implications in tumorigenesis and metastasis.

[37] Brizzi M. F., Tarone G., Defilippi P. (2012). Extracellular matrix, integrins, and growth factors as tailors of the stem cell niche.

[38] Pattabhi S. r., Martinez J. S., Keller T. C. S. (2014). Decellularized ECM effects on human mesenchymal stem cell stemness and differentiation.

[39] Xiong Y., He J., Zhang W., Zhou G., Cao Y., Liu W. (2015). Retention of the stemness of mouse adipose-derived stem cells by their expansion on human bone marrow stromal cell-derived extracellular matrix.

[40] Rakian R., Block T. J., Johnson S. M. (2015). Native extracellular matrix preserves mesenchymal stem cell “stemness” and differentiation potential under serum-free culture conditions.

[41] Antebi B., Zhang Z., Wang Y., Lu Z., Chen X. D., Ling J. (2015). Stromal-cell-derived extracellular matrix promotes the proliferation and retains the osteogenic differentiation capacity of mesenchymal stem cells on three-dimensional scaffolds.

[42] Zhang J., Li B., Wang J. H.-C. (2011). The role of engineered tendon matrix in the stemness of tendon stem cells in vitro and the promotion of tendon-like tissue formation in vivo.

[43] Lee J., Abdeen A. A., Kim A. S., Kilian K. A. (2015). Influence of biophysical parameters on maintaining the mesenchymal stem cell phenotype.

[44] Ansari S., Sarrion P., Hasani-Sadrabadi M. M., Aghaloo T., Wu B. M., Moshaverinia A. (2017). Regulation of the fate of dental-derived mesenchymal stem cells using engineered alginate-GelMA hydrogels.

[45] Rustad K. C., Wong V. W., Sorkin M. (2012). Enhancement of mesenchymal stem cell angiogenic capacity and stemness by a biomimetic hydrogel scaffold.

[46] Chien H.-W., Fu S.-W., Shih A.-Y., Tsai W.-B. (2014). Modulation of the stemness and osteogenic differentiation of human mesenchymal stem cells by controlling RGD concentrations of poly(carboxybetaine) hydrogel.

[47] McMurray R. J., Gadegaard N., Tsimbouri P. M. (2011). Nanoscale surfaces for the long-term maintenance of mesenchymal stem cell phenotype and multipotency.

[48] Zhao F., Veldhuis J. J., Duan Y. (2010). Low oxygen tension and synthetic nanogratings improve the uniformity and stemness of human mesenchymal stem cell layer.

[49] Park K. S., Ahn J., Kim J. Y., Park H., Kim H. O., Lee S. H. (2014). Poly-L-lysine increases the ex vivo expansion and erythroid differentiation of human hematopoietic stem cells, as well as erythroid enucleation efficacy.

[50] Heo J. S., Kim H. O., Song S. Y., Lew D. H., Choi Y., Kim S. (2016). Poly-L-lysine prevents senescence and augments growth in culturing mesenchymal stem cells ex vivo.

[51] Hofmeister L. H., Costa L., Balikov D. A. (2015). Patterned polymer matrix promotes stemness and cell-cell interaction of adult stem cells.

[52] Balikov D. A., Crowder S. W., Boire T. C. (2017). Tunable surface repellency maintains stemness and redox capacity of human mesenchymal stem cells.

[53] Nejati-Koshki K., Pilehvar-Soltanahmadi Y., Alizadeh E., Ebrahimi-Kalan A., Mortazavi Y., Zarghami N. (2017). Development of Emu oil-loaded PCL/collagen bioactive nanofibers for proliferation and stemness preservation of human adipose-derived stem cells: possible application in regenerative medicine.

[54] Pandolfi L., Furman N. T., Wang X. (2017). A nanofibrous electrospun patch to maintain human mesenchymal cell stemness.

[55] Pilehvar-Soltanahmadi Y., Nouri M., Martino M. M. (2017). Cytoprotection, proliferation and epidermal differentiation of adipose tissue-derived stem cells on emu oil based electrospun nanofibrous mat.

[56] Cheng N. C., Wang S., Young T. H. (2012). The influence of spheroid formation of human adipose-derived stem cells on chitosan films on stemness and differentiation capabilities.

[57] Taguchi T., Cho J. Y., Hao J., Nout-Lomas Y. S., Kang K. S., Griffon D. J. (2018). Influence of hypoxia on the stemness of umbilical cord matrix-derived mesenchymal stem cells cultured on chitosan films.

[58] Robinson J. L., McEnery M. A. P., Pearce H. (2016). Osteoinductive PolyHIPE foams as injectable bone grafts.

[59] Crowder S. W., Prasai D., Rath R. (2013). Three-dimensional graphene foams promote osteogenic differentiation of human mesenchymal stem cells.

[60] Lee J., Abdeen A. A., Kilian K. A. (2014). Rewiring mesenchymal stem cell lineage specification by switching the biophysical microenvironment.

[61] Olivares-Navarrete R., Lee E. M., Smith K. (2017). Substrate stiffness controls osteoblastic and condrocytic differentiation of mesenchymal stem cells without exogenous stimuli.

[62] Islam A., Younesi M., Mbimba T., Akkus O. (2016). Collagen substrate stiffness anisotropy affects cellular elongation, nuclear shape, and stem cell fate toward anisotropic tissue lineage.

[63] Lee S. H., Lee Y., Chun Y. W. (2014). In situ crosslinkable gelatin hydrogels for vasculogenic induction and delivery of mesenchymal stem cells.

[64] Zhang G., Drinnan C. T., Geuss L. R., Suggs L. J. (2010). Vascular differentiation of bone marrow stem cells is directed by a tunable three-dimensional matrix.

[65] Ross T. D., Coon B. G., Yun S. (2013). Integrins in mechanotransduction.

[66] Tay C. Y., Yu H., Pal M. (2010). Micropatterned matrix directs differentiation of human mesenchymal stem cells towards myocardial lineage.

[67] Lee J., Abdeen A. A., Zhang D., Kilian K. A. (2013). Directing stem cell fate on hydrogel substrates by controlling cell geometry, matrix mechanics and adhesion ligand composition.

[68] Alfaro M. P., Saraswati S., Young P. P. (2011). Chapter two - molecular mediators of mesenchymal stem cell biology.

[69] Wagner J., Kean T., Young R., Dennis J. E., Caplan A. I. (2009). Optimizing mesenchymal stem cell-based therapeutics.

[70] Balikov D. A., Fang B., Chun Y. W. (2016). Directing lineage specification of human mesenchymal stem cells by decoupling electrical stimulation and physical patterning on unmodified graphene.

[71] Oh S., Brammer K. S., Li Y. S. J. (2009). Stem cell fate dictated solely by altered nanotube dimension.

[72] Olivares-Navarrete R., Hyzy S. L., Hutton D. L. (2010). Direct and indirect effects of microstructured titanium substrates on the induction of mesenchymal stem cell differentiation towards the osteoblast lineage.

[73] Takahashi K., Yamanaka S. (2006). Induction of pluripotent stem cells from mouse embryonic and adult fibroblast cultures by defined factors.

[74] Wakayama T., Yanagimachi R. (1999). Cloning of male mice from adult tail-tip cells.

[75] Lister R., Pelizzola M., Kida Y. S. (2011). Hotspots of aberrant epigenomic reprogramming in human induced pluripotent stem cells.

[76] Kim D. S., Lee J. S., Leem J. W. (2010). Robust enhancement of neural differentiation from human ES and iPS cells regardless of their innate difference in differentiation propensity.

[77] Stadtfeld M., Apostolou E., Akutsu H. (2010). Aberrant silencing of imprinted genes on chromosome 12qF1 in mouse induced pluripotent stem cells.

[78] Nazor K. L., Altun G., Lynch C. (2012). Recurrent variations in DNA methylation in human pluripotent stem cells and their differentiated derivatives.

[79] Kaji K., Norrby K., Paca A., Mileikovsky M., Mohseni P., Woltjen K. (2009). Virus-free induction of pluripotency and subsequent excision of reprogramming factors.

[80] Woltjen K., Michael I. P., Mohseni P. (2009). *piggyBac* transposition reprograms fibroblasts to induced pluripotent stem cells.

[81] Okita K., Ichisaka T., Yamanaka S. (2007). Generation of germline-competent induced pluripotent stem cells.

[82] Nakagawa M., Koyanagi M., Tanabe K. (2008). Generation of induced pluripotent stem cells without Myc from mouse and human fibroblasts.

[83] Chakraborty S., Christoforou N., Fattahi A., Herzog R. W., Leong K. W. (2013). A robust strategy for negative selection of Cre-loxP recombination-based excision of transgenes in induced pluripotent stem cells.

[84] Stadtfeld M., Hochedlinger K. (2010). Induced pluripotency: history, mechanisms, and applications.

[85] Zhou W., Freed C. R. (2009). Adenoviral gene delivery can reprogram human fibroblasts to induced pluripotent stem cells.

[86] Zhang Y., Li W., Laurent T., Ding S. (2012). Small molecules, big roles – the chemical manipulation of stem cell fate and somatic cell reprogramming.

[87] Yu J., Hu K., Smuga-Otto K. (2009). Human induced pluripotent stem cells free of vector and transgene sequences.

[88] Ban H., Nishishita N., Fusaki N. (2011). Efficient generation of transgene-free human induced pluripotent stem cells (iPSCs) by temperature-sensitive Sendai virus vectors.

[89] Nakanishi M., Otsu M. (2012). Development of Sendai virus vectors and their potential applications in gene therapy and regenerative medicine.

[90] Shi Y., Desponts C., Do J. T., Hahm H. S., Scholer H. R., Ding S. (2008). Induction of pluripotent stem cells from mouse embryonic fibroblasts by Oct4 and Klf4 with small-molecule compounds.

[91] Kim E. J., Shim G., Kim K., Kwon I. C., Oh Y. K., Shim C. K. (2009). Hyaluronic acid complexed to biodegradable poly L-arginine for targeted delivery of siRNAs.

[92] Huangfu D., Maehr R., Guo W. (2008). Induction of pluripotent stem cells by defined factors is greatly improved by small-molecule compounds.

[93] Huangfu D., Osafune K., Maehr R. (2008). Induction of pluripotent stem cells from primary human fibroblasts with only *Oct4* and *Sox2*.

[94] Shi Y., Tae Do J., Desponts C., Hahm H. S., Schöler H. R., Ding S. (2008). A combined chemical and genetic approach for the generation of induced pluripotent stem cells.

[95] Ichida J. K., Blanchard J., Lam K. (2009). A small-molecule inhibitor of tgf-*β* signaling replaces *Sox2* in reprogramming by inducing *Nanog*.

[96] Maherali N., Hochedlinger K. (2009). Tgf*β* signal inhibition cooperates in the induction of iPSCs and replaces Sox2 and cMyc.

[97] Li W., Wei W., Zhu S. (2009). Generation of rat and human induced pluripotent stem cells by combining genetic reprogramming and chemical inhibitors.

[98] Trevisan M., Desole G., Costanzi G., Lavezzo E., Palu G., Barzon L. (2017). Reprogramming methods do not affect gene expression profile of human induced pluripotent stem cells.

[99] Planello A. C., Ji J., Sharma V. (2014). Aberrant DNA methylation reprogramming during induced pluripotent stem cell generation is dependent on the choice of reprogramming factors.

[100] Park J. H., Daheron L., Kantarci S., Lee B. S., Teixeira J. M. (2011). Human endometrial cells express elevated levels of pluripotent factors and are more amenable to reprogramming into induced pluripotent stem cells.

[101] Aasen T., Raya A., Barrero M. J. (2008). Efficient and rapid generation of induced pluripotent stem cells from human keratinocytes.

[102] Hanna J., Markoulaki S., Schorderet P. (2008). Direct reprogramming of terminally differentiated mature B lymphocytes to pluripotency.

[103] Aoi T., Yae K., Nakagawa M. (2008). Generation of pluripotent stem cells from adult mouse liver and stomach cells.

[104] Loh Y. H., Agarwal S., Park I. H. (2009). Generation of induced pluripotent stem cells from human blood.

[105] Li C., Zhou J., Shi G. (2009). Pluripotency can be rapidly and efficiently induced in human amniotic fluid-derived cells.

[106] Tamaoki N., Takahashi K., Tanaka T. (2010). Dental pulp cells for induced pluripotent stem cell banking.

[107] Ghosh Z., Wilson K. D., Wu Y., Hu S., Quertermous T., Wu J. C. (2010). Persistent donor cell gene expression among human induced pluripotent stem cells contributes to differences with human embryonic stem cells.

[108] Marchetto M. C. N., Yeo G. W., Kainohana O., Marsala M., Gage F. H., Muotri A. R. (2009). Transcriptional signature and memory retention of human-induced pluripotent stem cells.

[109] Kim K., Zhao R., Doi A. (2011). Donor cell type can influence the epigenome and differentiation potential of human induced pluripotent stem cells.

[110] Utikal J., Polo J. M., Stadtfeld M. (2009). Immortalization eliminates a roadblock during cellular reprogramming into iPS cells.

[111] Kim K., Doi A., Wen B. (2010). Epigenetic memory in induced pluripotent stem cells.

[112] Marion R. M., Strati K., Li H. (2009). A p53-mediated DNA damage response limits reprogramming to ensure iPS cell genomic integrity.

[113] Marion R. M., Strati K., Li H. (2009). Telomeres acquire embryonic stem cell characteristics in induced pluripotent stem cells.

[114] Stadtfeld M., Nagaya M., Utikal J., Weir G., Hochedlinger K. (2008). Induced pluripotent stem cells generated without viral integration.

[115] Sridharan R., Tchieu J., Mason M. J. (2009). Role of the murine reprogramming factors in the induction of pluripotency.

[116] Newman A. M., Cooper J. B. (2010). Lab-specific gene expression signatures in pluripotent stem cells.

[117] Chung T. L., Brena R. M., Kolle G. (2010). Vitamin C promotes widespread yet specific DNA demethylation of the epigenome in human embryonic stem cells.

[118] Chung T. L., Turner J. P., Thaker N. Y. (2010). Ascorbate promotes epigenetic activation of CD30 in human embryonic stem cells.

[119] Ohi Y., Qin H., Hong C. (2011). Incomplete DNA methylation underlies a transcriptional memory of somatic cells in human iPS cells.

[120] Singh N., Rahatekar S. S., Koziol K. K. K. (2013). Directing chondrogenesis of stem cells with specific blends of cellulose and silk.

[121] Cristallini C., Cibrario Rocchietti E., Accomasso L. (2014). The effect of bioartificial constructs that mimic myocardial structure and biomechanical properties on stem cell commitment towards cardiac lineage.

[122] Murphy C. M., Matsiko A., Haugh M. G., Gleeson J. P., O’Brien F. J. (2012). Mesenchymal stem cell fate is regulated by the composition and mechanical properties of collagen–glycosaminoglycan scaffolds.

[123] Mooney E., Mackle J. N., Blond D. J. P. (2012). The electrical stimulation of carbon nanotubes to provide a cardiomimetic cue to MSCs.

[124] Glennon-Alty L., Williams R., Dixon S., Murray P. (2013). Induction of mesenchymal stem cell chondrogenesis by polyacrylate substrates.

[125] Park I. H., Zhao R., West J. A. (2008). Reprogramming of human somatic cells to pluripotency with defined factors.

[126] Chou B. K., Mali P., Huang X. (2011). Efficient human iPS cell derivation by a non-integrating plasmid from blood cells with unique epigenetic and gene expression signatures.

[127] Jia F., Wilson K. D., Sun N. (2010). A nonviral minicircle vector for deriving human iPS cells.

[128] Warren L., Manos P. D., Ahfeldt T. (2010). Highly efficient reprogramming to pluripotency and directed differentiation of human cells with synthetic modified mRNA.

[129] Silva M., Daheron L., Hurley H. (2015). Generating iPSCs: translating cell reprogramming science into scalable and robust biomanufacturing strategies.

[130] Kim D., Kim C. H., Moon J. I. (2009). Generation of human induced pluripotent stem cells by direct delivery of reprogramming proteins.

[131] Zhou Q., Brown J., Kanarek A., Rajagopal J., Melton D. A. (2008). In vivo reprogramming of adult pancreatic exocrine cells to *β*-cells.

[132] Wolfrum K., Wang Y., Prigione A., Sperling K., Lehrach H., Adjaye J. (2010). The LARGE principle of cellular reprogramming: lost, acquired and retained gene expression in foreskin and amniotic fluid-derived human iPS cells.

[133] Dowey S. N., Huang X., Chou B. K., Ye Z., Cheng L. (2012). Generation of integration-free human induced pluripotent stem cells from postnatal blood mononuclear cells by plasmid vector expression.

[134] Streckfuss-Bomeke K., Wolf F., Azizian A. (2013). Comparative study of human-induced pluripotent stem cells derived from bone marrow cells, hair keratinocytes, and skin fibroblasts.

[135] Ieda M., Fu J. D., Delgado-Olguin P. (2010). Direct reprogramming of fibroblasts into functional cardiomyocytes by defined factors.

[136] Oda Y., Yoshimura Y., Ohnishi H. (2010). Induction of pluripotent stem cells from human third molar mesenchymal stromal cells.

[137] Vierbuchen T., Ostermeier A., Pang Z. P., Kokubu Y., Sudhof T. C., Wernig M. (2010). Direct conversion of fibroblasts to functional neurons by defined factors.

[138] Szabo E., Rampalli S., Risueno R. M. (2010). Direct conversion of human fibroblasts to multilineage blood progenitors.

[139] Huang P., He Z., Ji S. (2011). Induction of functional hepatocyte-like cells from mouse fibroblasts by defined factors.

[140] Stadtfeld M., Brennand K., Hochedlinger K. (2008). Reprogramming of pancreatic *β* cells into induced pluripotent stem cells.

